# Using a Design Thinking Approach to Develop a Social Media–Based Parenting Program for Parents of Children With Attention-Deficit/Hyperactivity Disorder: Mixed Methods Study

**DOI:** 10.2196/48201

**Published:** 2023-07-28

**Authors:** Umaporn Yam-Ubon, Therdpong Thongseiratch

**Affiliations:** 1 Department of Pediatrics, Faculty of Medicine, Prince of Songkla University, Songkhla, Thailand; 2 Songklanagarind ADHD Multidisciplinary Assessment and Care Team for Quality Improvement, Child Development Unit, Department of Pediatrics, Faculty of Medicine, Prince of Songkla University, Songkhla, Thailand

**Keywords:** attention-deficit/hyperactivity disorder, ADHD, parenting programs, human-centered design thinking, online interventions, COVID-19 pandemic, children, development, online parenting program, parenting, behavior, support, feasibility, social media, prototype, testing, design

## Abstract

**Background:**

Parenting programs have proven effective in improving the behavior of children with attention-deficit/hyperactivity disorder (ADHD). However, barriers such as job and transportation constraints hinder parents from attending face-to-face therapy appointments. The COVID-19 pandemic has further exacerbated these challenges.

**Objective:**

This study aimed to develop and test the feasibility of a social media–based parenting program for parents of children with ADHD, considering both the pre-existing challenges faced by parents and the additional barriers imposed by the COVID-19 pandemic.

**Methods:**

This study used a 5-stage design thinking process, encompassing empathizing with parents, defining their needs, ideating innovative solutions, prototyping the program, and testing the program with parents. Qualitative interviews were conducted with 18 parents of children with ADHD to understand their unique needs and values. Brainstorming techniques were used to generate creative ideas, leading to the creation of a prototype that was tested with 32 parents. Participants’ engagement with the program was measured, and posttraining feedback was collected to assess the program’s effectiveness.

**Results:**

Parents of children with ADHD encounter specific challenges, including managing impulsive behavior and difficulties in emotion regulation. The social media–based parenting program was delivered through the LINE app (Line Corporation) and consisted of 7 modules addressing topics related to ADHD management and effective parenting strategies. The program exhibited a high completion rate, with 84% (27/32) of participants successfully finishing it. Program provider–participant interaction peaked during the first week and gradually decreased over time. Qualitative feedback indicated that the program was feasible, accessible, and well received by participants. The LINE app was found to be convenient and helpful, and participants preferred content delivery once or twice per week, expressing acceptance for various content formats.

**Conclusions:**

This study emphasizes the significance of adopting a human-centered design thinking approach to develop parenting programs that cater to the unique needs and values of parents. By leveraging social media platforms, such as LINE, a parenting program can overcome the challenges posed by the COVID-19 pandemic and other constraints faced by parents. LINE offers a viable and feasible option for supporting parents of children with ADHD, with the potential for customization and widespread dissemination beyond the pandemic context.

## Introduction

Attention-deficit/hyperactivity disorder (ADHD) is a prevalent psychological condition among school-aged children, affecting approximately 7.2% of children and adolescents aged ≤18 years [1]. ADHD is associated with a range of negative consequences, including academic difficulties, impaired social relationships, and compromised quality of life, for both children and their families [2,3].

In the management of ADHD, parenting programs and medication are recognized as crucial components. Although medication can help manage symptoms, it is often insufficient to address the complex challenges associated with ADHD, without the support and guidance provided by parenting programs [4,5]. Parenting programs have shown effectiveness in reducing children’s disruptive behaviors and improving their adaptive functioning, making them a recommended primary intervention for managing ADHD symptoms [6,7]. These programs typically involve systematic interventions designed to educate and empower parents in effectively managing their child’s ADHD-related behaviors and challenges, with the ultimate goal of enhancing the child’s overall well-being [8,9].

Traditionally, parenting programs have been delivered through face-to-face group sessions, wherein parents from different families come together at a designated clinic or primary care unit [10]. Although some programs may offer in-home training, group-based face-to-face sessions have been the preferred format for managing ADHD symptoms. However, these programs encounter numerous structural barriers that limit their accessibility and impact. These barriers include financial constraints, limited resources, logistical challenges, time constraints, the lack of available childcare, and scheduling conflicts [11]. In addition to these structural barriers, there may be negative attitudes toward seeking mental health services for children, which can deter parents from engaging in group-based parenting programs [12,13].

The generalizability and effectiveness of traditional parenting programs may also be limited in low- and middle-income countries (LMICs), where socioeconomic conditions and health care systems differ significantly from those of high-income countries. Factors such as inadequate family income and limited access to mental health services can significantly moderate treatment outcomes [14]. Furthermore, the scarcity of trained mental health professionals in LMICs raises concerns about the feasibility and sustainability of therapist-delivered interventions [15]. Research on parenting programs in LMICs is relatively limited, and the delivery of such programs faces additional barriers, including the lack of a skilled and trained workforce and limited resources for supporting the implementation and dissemination of evidence-based interventions [16,17]. These challenges are further exacerbated by the ongoing COVID-19 pandemic, which has introduced additional barriers and limitations to the delivery of parenting programs.

The COVID-19 pandemic has disrupted many aspects of daily life, including access to health care services and support programs. Parents of children with ADHD have faced significant challenges in accessing and completing parenting programs during this time [18]. Lockdown measures and restrictions have resulted in increased behavioral problems among children with ADHD due to reduced opportunities for social interaction and disrupted routines [19]. Moreover, the pandemic has exacerbated parenting-related fatigue and psychological distress, further straining the well-being of parents [20]. Research has shown that parent support programs implemented during the COVID-19 pandemic have been associated with lower levels of parental stress and improved well-being [21].

To address the challenges faced by parents both during the COVID-19 pandemic and beyond, it is essential to prioritize the accessibility and effectiveness of parenting programs [22]. Social media–based programs have emerged as a promising solution, offering convenient and cost-effective interventions for various health issues, including obesity, diabetes mellitus, and certain mental health problems [23]. Social media–based programs provide higher accessibility rates than those of traditional face-to-face methods, allowing for timely intervention and support as soon as prodromal symptoms appear [24]. However, it is important to acknowledge that existing social media–based parenting programs have primarily been commercially driven [25], lacking effective bidirectional communication between parents and program providers [26-29], and have not undergone sufficient research to establish their efficacy in managing ADHD symptoms. Therefore, there is a need for evidence- and social media–based parenting programs specifically designed to address the unique challenges of managing ADHD.

## Methods

### Setting

This study, which is part of a social media–based parenting program project, took place at a large university hospital in Songkhla, Thailand, during June 2020 (the first wave of the COVID-19 pandemic in Thailand). A multidisciplinary team of health care professionals, including 1 general pediatrician, 2 developmental-behavioral pediatricians, 1 developmental-behavioral nurse, and 2 child psychologists, developed and coordinated the program.

### Ethics Approval

The study protocol was approved by the Human Research Ethics Committee of the Faculty of Medicine, Prince of Songkla University (research ethics committee number: 62-381-1-1).

### Study Design

We used a mixed methods approach, which involved participatory action research [30] and a design thinking process [31,32], to develop a social media–based parenting program for parents and caregivers of children with ADHD. The 5-step design thinking model from Stanford University’s Hasso-Plattner Institute of Design (d. school) was used, including empathy, define, ideate, prototype, and test [33,34].

#### Phase 1: Empathy

Semistructured in-depth interviews with parents and caregivers of children with ADHD were conducted to understand their challenges, problem-solving abilities, and preferences for a social media–based parenting program [35]. Demographic information was also collected.

#### Phase 2: Define

The research team synthesized the information from the interviews into a point-of-view statement [36], ensuring that it met the needs of the end users [35,37]. A group discussion refined the definition of parental requirements.

#### Phase 3: Ideate

Ideas for addressing the challenges and meeting the needs of parents were brainstormed, focusing on the creation of innovative solutions for the program content and features. Visual representations and structured brainstorming sessions facilitated the process [35,37].

#### Phase 4: Prototype

One or more prototypes were developed based on the previous phases’ data. Rapid feedback was collected during testing to determine end users' needs, and this feedback was used to further develop the prototype toward its full potential [35,37].

#### Phase 5: Test

Feedback from users was collected after the prototype’s launch [35,38]. Data on parental engagement and feedback on both benefits and potential negative interactions were used to refine the parent training program.

This study evaluated the five phases of the design thinking process, assigning distinct study populations to each stage based on the phase-related objectives. Data were collected from parents and caregivers of children with ADHD during the empathizing phase [39]. Afterward, during the ideation phase, these data were integrated into the initial program design and discussed with multidisciplinary health care professionals, including general pediatricians, developmental-behavioral pediatricians, developmental-behavioral nurses, and child psychologists. A prototype [38] was subsequently created and tested with parents and caregivers of children with ADHD. The participants in the test phase included those who were engaged in the empathizing phase, as well as new participants who were recruited specifically to increase the saturation of the findings of the testing phase. Posttraining feedback was collected, and participant engagement with the program was evaluated in the testing phase (Table 1).

**Table 1.** The five phases of the design thinking process and the methods used.

Phase	Concepts	Methods	Participants
Empathy	To discover users’ needs and values for a familial technology-based solution	In-depth interviews	18 parents
Define	To refine and narrow the definition in the revised solution to meed the end users' needs	Brainstorming technique	The research team
Ideate	To concentrate on idea generation and obtain innovative solutions for users	Brainstorming technique	The research team
Prototype	To generate the demonstrative solution for users	Design a web-based prototype for trial use	The research team
Test	To obtain pilot results and feedback on the prototype	Collect feedback via a quantitative and qualitative approach	32 parents

### Data Collection

To assess participant engagement, a manual text mining script was used to extract full transcripts of messages from moderators, researchers, and participants. These messages were categorized as text, images, videos, stickers, and audio messages. In addition to engagement, qualitative research was conducted during the empathy and test phases to gather data on parents’ needs, their perceptions, and lessons learned from the groups. Participants answered an open-ended questionnaire at the empathy phase and participated in in-depth interviews during the test phase.

### Data Analysis

The engagement analysis involved calculating the total number of messages per topic and the frequency of messages sent per week by mothers and moderators. The frequency of messages, according to the day of the week and time of the day, was also evaluated to estimate mothers’ most active moments in social media groups. In terms of the qualitative analysis, a grounded theory approach was adopted. The research team read all in-depth interview transcripts, identified emergent patterns, and used inductive coding to pinpoint key themes and concepts. Text was extracted from transcriptions to generate analysis content, themes, and findings, which were subsequently translated to English.

## Results

### Phase 1: Empathy

#### Participant Characteristics

A total of 18 participants (primarily mothers), with a mean age of 42 years, were enrolled in this phase. The demographic data can be found in Table 2. Over half of the participants (10/18, 56%) had a bachelor’s degree or higher. The qualitative research involved a thematic analysis and identified 8 themes, which are discussed below.

**Table 2.** Summary data of participants (N=18) in the empathy phase.

Characteristic		Value
Age (years), mean (range)		42 (25-55)
**Relationship, n (%)**		
	Father	4 (22)
	Mother	11 (61)
	Grandmother	2 (11)
	Aunt	1 (6)
**Education, n (%)**		
	Primary school	1 (6)
	High school	3 (17)
	Diploma	4 (22)
	Bachelor’s degree	7 (39)
	Master’s degree	3 (17)
**Occupation, n (%)**		
	Government official	4 (22)
	Businessman	2 (11)
	Freelance	6 (33)
	Gardener	2 (11)
	Merchant	3 (17)
	Housewife	1 (6)

#### Theme 1: Parenting Problems

Participants expressed concerns about their children’s behavior and its impact on their own mental health (eg, “Not only is unfinished homework the most common childrearing issue, but parents' mental health is also important in childrearing management”).

#### Theme 2: Parental Needs

Parents expressed a desire to learn techniques to help their children academically and emotionally (eg, “I’d like to learn the technique so that I can help him improve his academic performance”).

#### Theme 3: Target Problems to Resolve

Participants identified their children’s primary issues, such as a lack of focus, disobedience, and a lack of self-control (eg, “I wish he was focused on my words, obedient, and capable of self-control”).

#### Theme 4: Internet Use Behavior

Parents shared information about their preferred electronic devices and social media–based platforms (eg, “I have a smartphone and a PC. In my daily existence, I frequently utilize LINE, Facebook, and Google”).

#### Theme 5: Frequency, Duration, and Time Periods

Participants discussed their daily internet usage habits and preferred times for intervention (eg, “I use my cellphone for 1–2 hours a day, between the hours of 8 and 10p.m.”).

#### Theme 6: Media Types

Parents indicated their preferred media formats for the parenting program, such as infographics and videos (eg, “I recommend infographics and brief video clips”).

#### Theme 7: Individual Versus Group Counseling

Participants debated the merits of individual versus group counseling for addressing parenting issues (eg, “If the program is set up in a group setting, we may share experiences and track the development of the children”).

#### Theme 8: Target Recipients

Parents highlighted the importance of involving all family members in the parenting intervention (eg, “Information should be sent to the parents, grandparents, and grandfather due to possible inconsistencies in childrearing within the family”).

### Phase 2: Define

In this phase, the research team, consisting of developmental-behavioral pediatricians, general pediatricians, a developmental-behavioral pediatrics nurse, and a psychologist, aimed to incorporate user requirements from the previous phase into the problem statement. They identified the users as “parents and caregivers of children with ADHD” and determined their critical need to be “learning parenting techniques” to help ameliorate their children’s behavioral problems.

Through the analysis of the empathize phase, the team gained real insights into the parents’ and caregivers’ perspectives. Parents expressed feelings of being overwhelmed and sometimes felt helpless in the face of their children’s ADHD-related challenges. They also emphasized the importance of practical and easily applicable solutions that could seamlessly be integrated into their daily routines. The point-of-view statement, which was formulated based on these insights, was as follows:

Parents and caregivers of children with ADHD need effective parenting techniques and support to improve their children’s behavior and overall well-being, while addressing their own emotional needs and fostering a sense of empowerment.

The insight synthesized from the data denoted the desire “to improve the behavioral issues of children with ADHD.” This understanding led to the formulation of a “How Might We” (HMW) statement, which serves as a driving question to inspire innovative solutions. The HMW statement developed was as follows:

How might we create a supportive and accessible program that equips parents and caregivers of children with ADHD with the necessary parenting techniques to effectively manage their children’s behavioral challenges and improve their overall well-being, while also addressing their emotional needs and fostering a sense of empowerment?

### Phase 3: Ideate

The ideation phase began with the development team discussing potential solutions, using the HMW statement as a guide. The facilitator led a roundtable discussion centered around the HMW question.

To ensure a diverse range of ideas during the brainstorming session, the team used the “Work Alone Together” technique. This method allowed individual team members to generate ideas independently before sharing them with the group. This approach fostered creativity and encouraged the team to explore multiple channels, formats, and platforms for the parenting program, such as using social media, videoconferencing, booklets, podcasts, and group meetings to deliver the program content.

After the brainstorming session, the team used an affinity map (Textbox 1) to categorize and organize the generated ideas. This process allowed them to identify similarities, relationships, and patterns among the concepts. The affinity map included categories like LINE (Line Corporation), Facebook (Meta Platforms Inc), YouTube (YouTube LLC) or videos, videoconferences, booklets, lists, group meetings, telephone counseling, e-counseling, podcasts, behavioral tutorials, and television advertising.

Textbox 1. **Affinity mapping.**

1. LINE (Line Corporation)
  - LINE group
  - Parent LINE group
  - LINE group: parents and teacher
  - Individual LINE
2. Facebook (Meta Platforms Inc)
  - Facebook: changing behavior
  - Facebook page for children with attention-deficit/hyperactivity disorder (ADHD)
  - Facebook pages
  - Facebook group of ADHD caregivers
3. YouTube (YouTube LLC) or videos
  - Suggested list of videos in YouTube
  - Develop video scenarios for behavioral management (published via a YouTube channel)
  - Videos describing common behavioral problems
4. Videoconferences
  - Use Zoom (Zoom Video Communications Inc) program for tracking behavioral problems
5. Booklets
  - Booklet for behavioral management
  - Brochure
  - Question-and-answer brochure about common behavioral problems
6. Lists
  - List of interesting Facebook pages
  - Lists of websites about parenting
  - Suggest websites about ADHD
7. Group meetings
  - Group meeting for ADHD caregivers
  - Parent group meeting
8. Telephone counseling
9. e-Counseling
  - Advise behavioral management
  - Target behavior according to age
10. Podcast: parenting management
11. Behavioral tutorial via teacher
12. Television advertising at schools and hospitals

**Figure 1.**
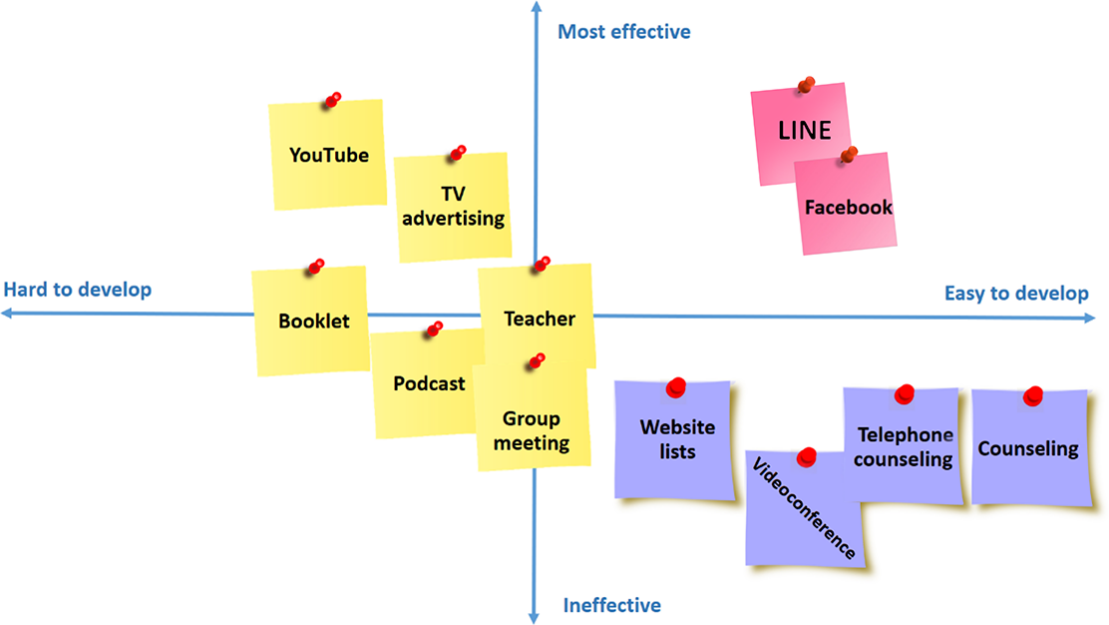
Prioritization map.

The team used a prioritization map (Figure 1) to rank the ideas. Before using the member voting technique, they arranged the ideas in the four quadrants of the prioritization map. This approach helped them evaluate the importance and feasibility of each idea. After organizing the ideas in the quadrants, the team voted to determine the most effective and straightforward platform for developing the parent training program. The results indicated that LINE was the preferred choice. Consequently, LINE was chosen as the social media platform for content delivery in this study. The ideation phase also resulted in the creation of prototypes for the proposed parent training programs, which included text messages, infographics, videos, and assignments. Weekly learning goals were set, and samples of content for end users were designed.

### Phase 4: Prototype

The prototype phase involved the development of a comprehensive social media–based parent training program, based on the insights and ideas gathered in the previous three phases of the design thinking process. The prototype consisted of a LINE-based parenting program, which included various components, such as content creation, user experience (UX) development of infographics, and video clip production, to provide parenting models for parents and caregivers.

#### Content Prototype

The content prototype was designed to address the needs and preferences of the target users. The team developed a curriculum of 7 parent training modules, which were to be completed over a 7-week intervention period. Each module covered a specific topic related to parenting children with ADHD and offered practical strategies, advice, and tips. The content was delivered through a combination of text messages, infographics, videos, and assignments, ensuring that the program was engaging, informative, and easy to follow (Table 3).

**Table 3.** Prototype LINE-based parenting contents.

Weeks and days		Contents
**Weeks 1-2**		Introduction
	Day 1	Program introduction
	Days 1-2	Understanding ADHD^a^
	Day 3	Treatment of ADHD
	Day 4	Case scenario video (homework)
	Day 5	Parental concern and setting goal
	Day 6	Rationale, rule, and cycle of problem
	Days 6-7	Basic principles in child behavioral management
	Day 8	Advantage of parent training
	Days 9-10	ABC^b^ model (homework)
	Days 11-12	Problem solutions
	Days 13-14	Summary and discussion
**Week 3**		Basic communication skills
	Day 1	Inappropriate conversations (homework)
	Days 1-2	Negative conversations
	Days 3-4	Using “I” messages and “you/he/she” messages
	Day 5	Effective communication
	Day 6	Constructive instruction
	Day 7	Case scenario video (homework)
**Week 4**		Praise
	Day 1	Praise
	Day 2	Case scenario video (homework)
	Day 3	Principles, components, and examples
	Days 3-6	Diary notes
	Days 5-7	Reminders about basic communication skills
	Day 7	Reflection
**Week 5**		Rewards
	Day 1	Types of rewards
	Day 2	Principles of rewards
	Day 3	Define target behaviors
	Days 4-6	Reflection and technique suggestion
	Days 4-6	Diary notes
	Day 7	Summary and discussion
	Day 7	Reminders about previous skills
**Week 6**		How to deal with behavioral problems
	Day 1	Objectives and principles of behavioral management
	Day 2	Effective behavioral management
	Day 3	Time-out
	Day 4	Verbal command
	Day 4	Ignorance
	Days 5-6	Case scenario video (homework)
	Days 2-6	Sharing the experiences
	Days 2-6	Diary notes
	Day 7	Summary and discussion
**Week 7**		How to deal with homework
	Day 1	Principles of doing homework
	Day 2	How to deal with homework
	Day 3	Behavioral management
	Day 4	Self-reporting card
	Days 2-7	Sharing the experiences
	Days 2-7	Diary notes
	Day 7	Summary and discussion

a ADHD: attention-deficit/hyperactivity disorder.

b ABC: Antecedent, Behavior, and Consequences.

#### UX Development and Parent Interaction

**Figure 2.**
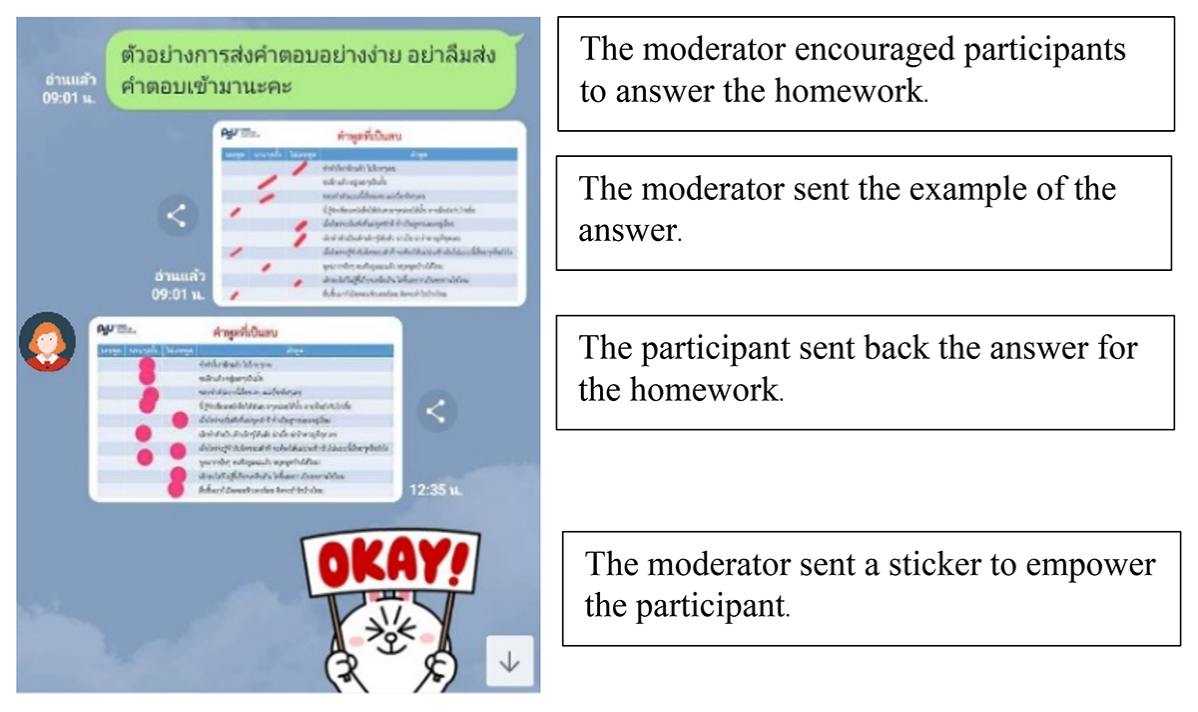
Example of a conversation through a moderated LINE group.

In order to create a visually appealing, user-friendly experience that also facilitated meaningful interaction among parents, the team focused on the design of infographics and on fostering engagement among users. The infographics were designed to be clear, concise, and informative, presenting complex information in an easily digestible format. They included visuals, charts, and graphs to illustrate key points and concepts, making it easier for parents to understand and apply the strategies discussed in the program. To promote interaction and engagement, the team incorporated features that encouraged parents to share their experiences, ask questions, and provide support to one another. This was achieved through the creation of a dedicated LINE group, wherein users could engage in discussions, share their progress, and seek advice from both experts and fellow parents. The group also served as a platform for sharing additional resources, hosting live question-and-answer sessions, and conducting polls to gather feedback and gauge user satisfaction. By fostering a sense of community and providing opportunities for interaction, the UX development aimed to enhance the overall effectiveness and appeal of the program (Figure 2).

#### Video Clip Production

To further enhance the program’s effectiveness and user engagement, the team produced a series of video clips that demonstrated various parenting models and techniques. These videos featured expert advice, real-life examples, and step-by-step instructions to help parents and caregivers better understand and apply the strategies discussed in the program. The videos were designed to be short, focused, and easy to access, allowing users to watch them at their convenience.

#### Prototype Evaluation

Once the initial prototype was complete, it was evaluated by a group of parents and caregivers who participated in a pilot testing phase. Participants completed a web-based eligibility screening and informed consent process as part of the baseline survey. Those who met the eligibility criteria and provided their consent were directed to complete comprehensive baseline surveys. Participants received a general schedule for the completion of the seven parent training modules and were provided with approximately one or two inputs daily, between 6 PM and 9 PM. They were also given 2 assignments per week, with a 1-week deadline for completing the assigned tasks.

The feedback gathered from the pilot testing phase was used to refine and improve the prototype, ensuring that it effectively addressed the needs and preferences of the target users. The final prototype of the LINE-based parenting program was then prepared for implementation and further evaluation.

### Phase 5: Test

#### Participants

A total of 32 participants from 24 families, who were parents or caregivers of children (aged 4 to 10 years) diagnosed with ADHD, were enrolled in this phase. Participants included mothers (20/32, 63%) and primary caregivers (29/32, 91%) with a bachelor’s degree. Most children were boys aged 8 to 10 years who were receiving medication as primary treatment (24/32, 75%).

#### Participant Engagement in the Program

Engagement was assessed by tracking the content marked as “read” in the LINE app every week over the 7-week intervention period. The responses to interventions were categorized on a 4-point scale (0=unread; 1=only read; 2=interaction with the intervention [posting a sticker, responding with “OK,” or writing “thank you”]; 3=initiating a discussion or asking a question). A score of 46 represented that the participant had read all the content, while scores above 46 indicated that they read and interacted with the program providers. Table 4 illustrates the distribution of participants’ levels of engagement with the program, showing that the majority of them (16/32, 50%) completed the intervention, while 34% (11/32) demonstrated higher engagement by actively interacting with the content. On the other hand, 16% (5/32) of the participants were classified as nonadherent, indicating a lower level of engagement with the program.

**Table 4.** Classification of patient engagement scores.

Classification	Score	Participants (N=32), n (%)
Nonadherence	<46	5 (16)
Completion	46	16 (50)
Adherence	>46	11 (34)

**Figure 3.**
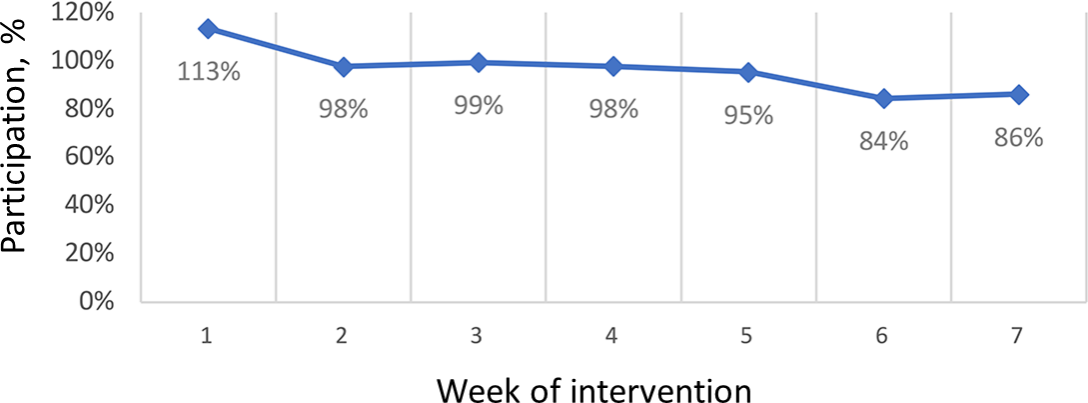
Response from intervention.

Participants’ engagement levels were charted by using a linear graph, which depicted their levels of interaction with the content over the 7-week period (Figure 3). The first week showed the highest engagement levels, which gradually decreased over time, reaching the lowest point in the last week.

These data highlight the overall participant engagement in the program and how it evolved throughout the intervention.

### Posttraining Feedback

Qualitative data were collected from 32 participants via in-depth interviews after they completed the LINE parenting program. The following four themes emerged from the thematic analysis: (1) social media platform, (2) internet use behavior, (3) content formats, and (4) intervention adherence.

#### Theme 1: Social Media Platform

Participants found the LINE app to be convenient and accessible, accessing it daily. They appreciated being able to save and review images and videos. One participant said:

LINE is convenient, but an OPD visit is still necessary because when the doctor speaks to my son, he listens and follows your advice.

#### Theme 2: Internet Use Behavior

Participants often read the content at night or on weekends, and the daily delivery of content was considered appropriate. Primary caregivers preferred daily content delivery, while others favored weekly delivery. A participant stated:

I read the content on weekdays, after 21:00. Frequency of content delivery is acceptable at about 2–3 times per day.

#### Theme 3: Content Formats

All content formats were deemed acceptable, and participants found Google Forms (Google LLC) to be user-friendly for assignments and questionnaires. They suggested the addition of more video clips that demonstrate communication techniques. One participant made the following suggestion:

Google forms make it simple to complete assignments and questionnaires. Some content should be adjusted to reflect the child’s age and developmental stage.

#### Theme 4: Intervention Adherence

Participants applied the praising and communication skills from the modules in their daily lives. Many believed that a parenting program is suitable for primary caregivers, though some recommended adaptations for specific situations. For example, a participant said:

A parenting program is appropriate for primary caregivers. If the recipient is not the primary caregiver, the content should be delivered at a slower rate.

## Discussion

### Principal Findings

The innovative social media–based parenting program, which was developed through a design thinking process, demonstrates the potential of leveraging familiar and accessible platforms to address the needs of parents and caregivers of children with ADHD. By using the LINE app, the program delivered content in varied formats, offering valuable ADHD information, parenting guidance, assignments, and screen-to-screen consultations. This approach made the program accessible, feasible, and acceptable for Thai parents, who found it convenient to save and review information as needed. The success of the program can be attributed to the incorporation of the participants’ needs and values, its feasibility, and its acceptability, as evidenced by the high retention rate and qualitative feedback.

The internet use behaviors observed in this study, such as preferred communication times and the use of familiar social media apps, align with previous research [40-45]. This highlights the importance of considering user preferences and behaviors in the development of social media–based interventions, as doing so can increase engagement, effectiveness, and acceptability. The use of the LINE app facilitated user engagement, as it was familiar, simple, and accessible. This supports the notion that promoting credible social media sites and educating users on proper social media use can help reduce misinformation regarding parenting issues [46-50]. Additionally, using a platform that users are familiar with can minimize barriers to access and improve UX.

The content, structure, and delivery of the program were crucial factors that contributed to its feasibility and acceptability. Participants appreciated the convenience of receiving the program through the LINE app, which they used daily. The praise-focused content was particularly interesting to the participants, in line with a previous meta-analysis that found a large effect size for praise, reward, and logical sequence techniques [8]. The program’s flexible structure allowed parents to engage with the content at their preferred times and facilitated the sharing of content with other individuals facing similar challenges.

Participants were highly engaged with the program, with 84% (27/32) of participants expressing interest in the intervention. Parents were familiar with the LINE app and discussed their children’s behavioral difficulties. Although participants did not always respond immediately to the inputs, they reported reading the material later, saving photographs and video clips on their devices, and sharing the materials with others who faced similar problems with their children’s behavior. These engagement rates compare favorably with previous studies; Franke et al [51] found that 55% of participants completed all 8 modules of their intervention, while Baker et al [52] reported a retention rate of approximately 92.5% at postintervention assessment, with 81% of participants completing the 9-month follow-up evaluation.

Participants believed that the program should also include other caregivers, but the content should be brief and summarized for nonprimary caregivers. Previous research has also shown that interactive programs are more effective in improving child behavior than noninteractive programs [47]. This highlights the importance of customization in social media–based parenting programs. Participants also provided valuable suggestions for improving the program, such as including more behavioral management video simulations and tailoring content to different family circumstances. These recommendations highlight the importance of continuously refining and adapting interventions to better address the specific needs and contexts of target populations. Customization and personalization have been shown to be significant factors in the success of social media–based parenting programs [8,9,53,54].

### Future Research and Implementation

With regard to future research and implementation, human-centered solutions are necessary to reduce the gap between parents’ needs and the content and structure of parenting programs, particularly for parents of children with ADHD. Future studies should focus on personalization, engagement, and positive parental experiences to improve social media–based parenting programs. Additionally, more research is needed to understand how to optimize the integration of technology into parenting interventions, especially in LMICs, where resources and access to quality information may be limited. Collaboration among health care providers, researchers, and technology developers is essential for the creation of effective and accessible social media–based interventions. By working together, these stakeholders can ensure that evidence-based information and guidance are disseminated to parents and caregivers in an engaging, user-friendly format. Furthermore, public health organizations and educational institutions can play a critical role in promoting and supporting the adoption of such interventions within communities.

### Limitations

This study has several limitations that should be acknowledged. First, the sample size was small, which may impact the statistical power of the quantitative findings. However, it is important to note that this study used a mixed methods approach, which allows for data saturation and ensures comprehensive exploration of the research questions beyond mere statistical power. Second, this study was conducted within a single institution; as such, there is a possibility of selection bias, and the findings many not be generalizable to a broader population. To address this limitation, future research should consider multicenter collaborations to include more diverse settings and participants, thereby enhancing the external validity of the findings. Additionally, volunteer bias may have influenced the results, as participants who volunteered for this study may have had characteristics or motivations that were distinct from those of nonparticipants. To mitigate this bias, future studies could explore recruitment strategies that reach a wider range of participants, aiming for a more representative sample.

### Conclusion

This study provides evidence for the feasibility and acceptability of a social media–based parenting program for Thai parents of children with ADHD. The program was developed by using a design thinking approach and delivered through the LINE app, a social media platform that was familiar and accessible to the participants. The program provided valuable technical skills related to nurturing children with ADHD and was well received by parents and caregivers. Future research on social media–based parenting programs should focus on personalization and on meeting the specific needs of parents and caregivers to improve long-term outcomes for children with ADHD and their families.

## Abbreviations

ADHD: attention-deficit/hyperactivity disorder
HMW: How Might We
LMIC: low- and middle-income country
UX: user experience
